# Thermally-Induced Deformations and Warpages of Flip-Chip and 2.5D IC Packages Measured by Strain Gauges

**DOI:** 10.3390/ma14133723

**Published:** 2021-07-02

**Authors:** Ming-Yi Tsai, Yu-Wen Wang, Chia-Ming Liu

**Affiliations:** Department of Mechanical Engineering, Chang Gung University, Kwei-Shan, Tao-Yuan 333, Taiwan; wanglovedog@gmail.com (Y.-W.W.); s3556947@gmail.com (C.-M.L.)

**Keywords:** flip-chip package, 2.5D package, thermal warpage, strain gauge

## Abstract

The thermal warpage problems in integrated circuit (IC) packaging exist in both flip-chip and two-and-a-half dimensional integrated circuits (2.5D IC) packages during manufacturing processes and thermal cycling service. This study proposes a simple and easy-to-use strain gauge measurement associated with a beam model theory to determine the thermally induced deformations and warpages of both packages. First, validation and limitations of the beam model theory are presented. Then, the thermally induced out-of-plane deformations for both packages are well described by the finite element method (FEM) simulation with a good consistency to full-field shadow moiré experimental results. The strain gauge measurements were implemented experimentally, and the thermal strain results were found to be well consistent with validated FEM ones. As a result, out-of-plane thermal deformations and warpages of the packages, calculated from the beam model theory with extracted curvature data from the strain gauge, were in reasonably good agreement with those from FEM analysis and shadow moiré measurements. Therefore, the strain gauge method of featuring point strain measurement combined with the beam model theory proved feasible in determining the thermal deformations and warpages of both IC packages.

## 1. Introduction

**Figure 1:**
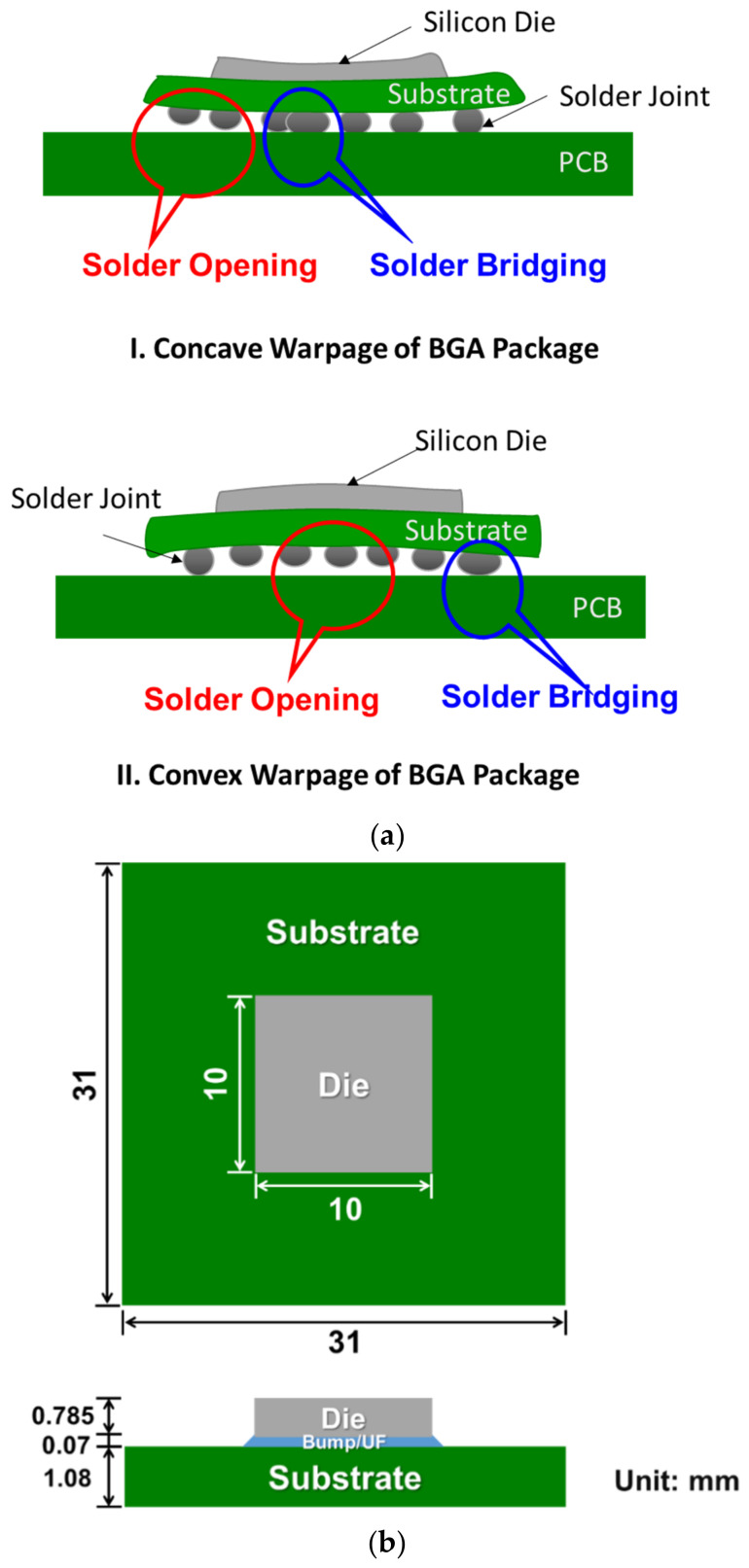

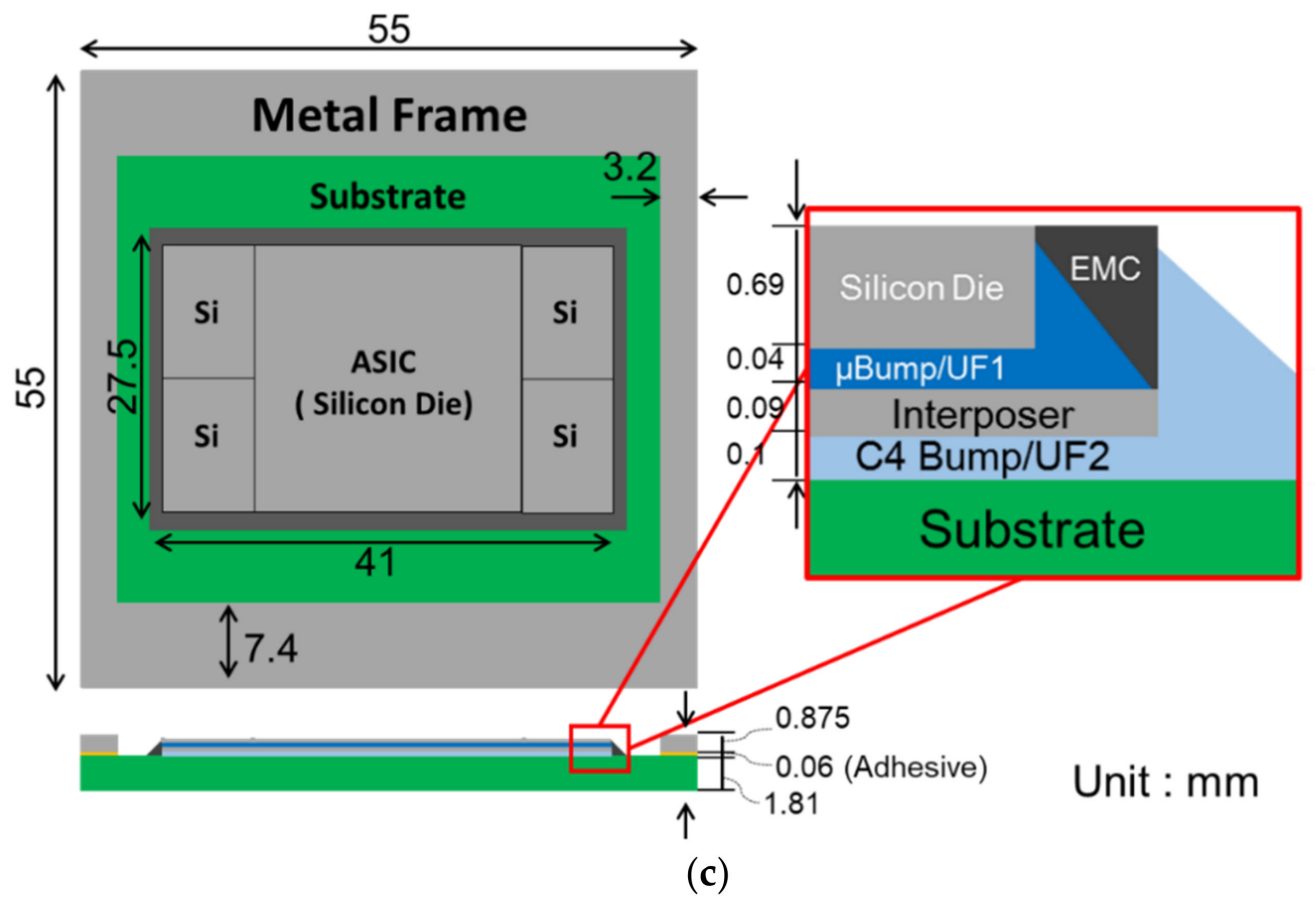
Schematics of (**a**) thermal warpage induced solder joint defects, (**b**) a flip-chip package and (**c**) a 2.5D IC package with detailed materials and dimensions.

The flip-chip packages and the two-and-a-half dimensional integrated circuits (2.5D IC) packages are popularly used in advanced integrated circuits (IC) packaging [1,2,3]. However, those packages suffer from thermal warpage problems, which may cause solder joint defects, shown in Figure 1a, during manufacturing processes and thermal cycling service due to mismatches of coefficients of thermal expansion (CTE) between the inherent materials [3,4,5,6,7]. Unlike the conventional flip-chip packages, which use a silicon chip to directly bond to the substrate, the 2.5D IC packaging technology uses an additional silicon interposer as a platform to interconnect and integrate heterogeneous or homogeneous chips horizontally and vertically before flip-chip bonding to the substrate [1,3]. Such technology gained more attention in advanced IC packaging for heterogeneous integrations recently. For strain measurement, electrical resistance strain gauges were widely used for point (or local) mechanical strain measurements for more than a century in various engineering applications [8]. Its advantage over other full-field optical methods such as shadow moiré [4,9,10], Twyman–Green interferometry [9,11], or digital image correlation (DIC) [12,13] is that it is easy to use and can provide in-situ and real-time thermal strain measurements, especially for complex printed circuit boards (PCB) during the heating at a solder reflow oven [14]. Some preliminary results of thermal deformations of the 2.5D IC package were presented in an international conference [15]. In this study, the conventional strain gauges were employed for measuring the curvatures of the flip-chip package and the 2.5D IC package reinforced with a metal frame (as shown in Figure 1b,c) under thermal loads. Then, those curvature data were converted to deformations (or warpages) data using a beam model theory. Note that the thermal deformation of the package here is presented by its out-of-plane displacement referred to fixed center point, while the warpage is the out-of-plane displacement of a certain point. Feasibility and validity of this strain gauge method are thoroughly discussed herein by comparing the obtained thermal deformation and warpage results with those from the full-field shadow moiré and the FEM analysis in this study.

## 2. Methodologies

The methods used in this study for determining the thermal deformations and warpages of the IC packages are briefly illustrated in this section, including the combination of strain gauge and a beam model theory, the finite element analysis, and a shadow moiré measurement.

### 2.1. Deformation Measurement by Stain Gauges Associated with A Beam Model

The typical strain gauge measurement [8] can be described as

(1) $$\varepsilon_{a}=\varepsilon_{t}+\frac{\left(\gamma_{g}+\gamma_{w}\right)}{S_{g}}\Delta T$$

where *ε_a_* is an apparent strain which is directly obtained from strain gauge measurement system, *ε_t_* is a true strain which is an actual strain on the measured point of the specimen, *S_g_* is a gauge factor, Δ*T* is thermal loading, and *γ_g_* and *γ_w_* are the temperature coefficients of resistivity of gauge-grid metal material and connected lead wire, respectively. Back-to-back strain gauges were adhered at a specific position on both top and bottom surfaces of the IC package for measuring the thermal strains on the package during heating and cooling processes. The apparent strains on the top and the bottom surfaces (*ε_a,top_* and *ε_a,bot_*) of the package can be written individually in terms of the true strains on the top and the bottom surfaces (*ε_t,top_* and *ε_t,bot_*) as

(2) $$\varepsilon_{a,top}=\varepsilon_{t,top}+\frac{\left(\gamma_{g}+\gamma_{w}\right)}{S_{g}}\Delta T$$

(3) $$\varepsilon_{a,bot}=\varepsilon_{t,bot}+\frac{\left(\gamma_{g}+\gamma_{w}\right)}{S_{g}}\Delta T$$

The strain data at various temperatures could be further converted into bending strains (*ε_b_*) and bending curvatures (*k*) of the IC package by Equations (4) and (5), respectively. Then, the out-of-plane displacement (deformation) of the IC package under different temperatures could be further calculated by Equation (6) with a given curvature and distance *x* from the center.

(4) $$\varepsilon_{b}=(\varepsilon_{a,bot}-\varepsilon_{a,top})/2=(\varepsilon_{t,bot}-\varepsilon_{t,top})/2$$

(5) $$k=2\varepsilon_{b}/t=(\varepsilon_{t,bot}-\varepsilon_{t,top})/t$$

(6) $$W=kx^{2}/2$$

where *t* is the package thickness, and *W* is the out-of-plane displacement (deformation) at the given distance *x* with a constant curvature *k*.

**Figure 2:**
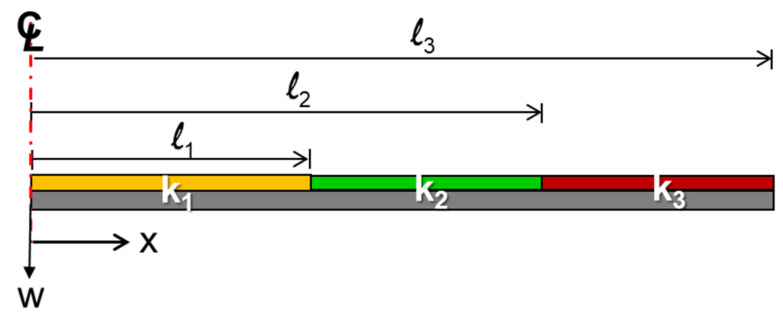
Schematic of the beam with constant curvatures k_1_, k_2_, and k_3_ at different segments.

Since the curvature of the package may not be constant across the entire surface of the package, a beam model theory, as shown in Figure 2, with multiple curvatures for the out-of-plane displacement calculation is proposed in this study. This model is based on a beam theory with an assumption of a small deflection. The *k_i_* is a constant curvature on the segment *i* between the length *l*_*i*−1_ and *l_i_*. The *k*_1_ represents the curvature of the center segment. The associated equations of an out-of-plane displacement (or deflection) *W_i_* within the segment *i* are listed [14] as follows:

*W*_0_ = 0, *x* = 0 (7a)

(7b) $$W_{1}(x)=\frac{1}{2}k_{1}x^{2},\;0\leq x\leq l_{1}$$

(7c) $$W_{2}(x)=W_{1}(l_{1})+k_{1}l_{1}(x-l_{1})+\frac{1}{2}k_{2}{\left(x-l_{1}\right)}^{2},\;l_{1}\leq x\leq l_{2}$$

(7d) $$W_{3}(x)=W_{2}(l_{2})+[k_{1}l_{1}+k_{2}(l_{2}-l_{1})](x-l_{2})+\frac{1}{2}k_{3}{\left(x-l_{2}\right)}^{2},\;l_{2}\leq x\leq l_{3}$$

### 2.2. FEM Simulation

**Figure 3:**
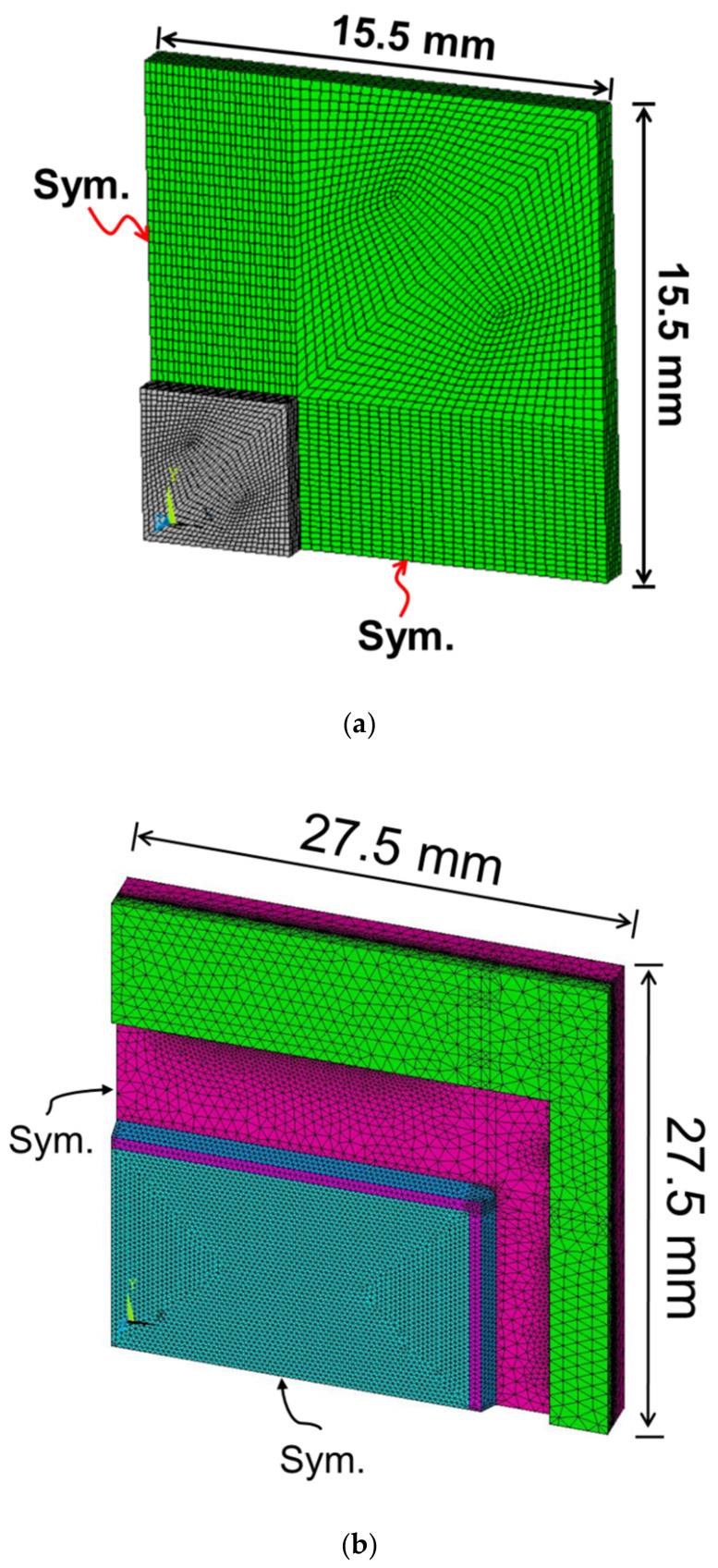
A quarter model and meshes of (**a**) a flip-chip package and (**b**) a 2.5D IC package used in the finite element analysis.

**Figure 4:**
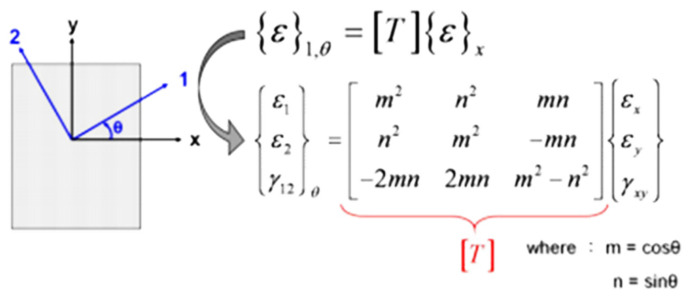
Transformation of strain vector {*ε*}_*x*_ from the x–y coordinate to {*ε*}_1,*θ*_ in the 1–2 coordinate with a rotation angle of *θ*.

An isothermal linear FEM analysis was performed to calculate the thermally induced package deformation and warpage due to the CTE mismatch between the inherent materials properties. The schematics of the FEM models are also shown in Figure 3a,b for the flip-chip and the 2.5D IC packages, respectively, with related boundary conditions and meshes in a quarter model. Material mechanical properties used in the finite element analysis are shown in Table 1, in which most data are provided by material vendors. Since the obtained strain data from the FEM analysis were in the x–y coordinate, the transformation of strain vector {*ε*}_*x*_ from the x-y coordinate to {*ε*}_1,__*θ*_ in the 1–2 coordinate with a rotation angle of *θ* was needed during the data process and is illustrated in Figure 4, in which [T] is a transformation matrix as a function of *θ*. It is noted that this strain transformation was used for calculating a normal (or axial) strain in the 1-axis direction, such as the diagonal direction of the package or the strain gauge direction during the data process. Then, the FEM simulation results were further used to compare with those from strain gauge measurements in terms of curvatures and from moiré measurement in terms of deformations for both packages. In addition, there were two 2D FEM models—plane stress and axisymmetric models—implemented in this study for validating the beam model theory.

### 2.3. Shadow Moiré Measurements

The shadow moiré method [4,9,10] is widely used to measure the out-of-plane displacement of the specimens. The systems with the sensitivity of 12.7 μm/fringe and 25.4 μm/fringe were used for the thermal deformation measurement of the flip-chip and the 2.5D IC packages, respectively. An oven or a hot plate were used to heat the specimens and provided thermal loading from room temperature to 260 °C. The present thermal cycling test plus data recording in general took about one hour from room temperature to 260 °C and longer for cooling by natural cooling. Two test samples, a flip-chip package with a size of 31 × 31× 1.94 mm^3^ and a 2.5D IC package with a size of 55 × 55 × 2.73 mm^3^ (shown in Figure 1b,c), were tested in the moiré experiments by measuring surfaces on their substrates in this study.

## 3. Results and Discussion

The obtained results of the flip-chip package and the 2.5D IC package are extensively discussed individually in this section, including the beam model verification, the strain gauge measurement, the moiré measurement, and the FEM analysis.

### The Case of Flip-Chip Package

(a) Validation of beam model with FEM simulation

**Figure 5:**
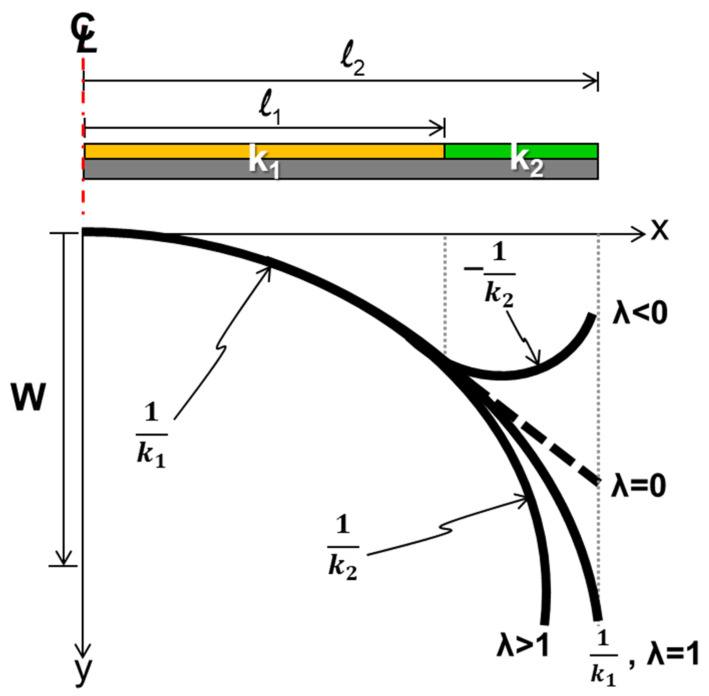
Schematic of the out-of-plane displacement of the beam with given constant curvatures *k*_1_ and various *k*_2_ (or various λ = *k_2_/k_1_*).

A beam with two curvatures (*k*_1_ and *k*_2_) over the entire length was considered. A schematic of the out-of-plane displacements of the beam with given constant curvatures *k*_1_ and various *k*_2_ is shown in Figure 5. The normalized curvature was *λ* = *k*_2_/*k*_1_ and the normalized beam length was *β* = *l*_2_/*l*_1_. From the beam theory (Equations (7b) and (7c)), *W*_2_(*l*_2_)_λ_ = _1_ and *W*_2_(*l*_2_)_*λ*_, representing the out-of-displacements (warpages) for *λ* = 1 and any value, respectively, at *x* = *l*_2_, could be described as follows:

(8) $$W_{2}{\left(l_{2}\right)}_{\lambda =1}=\frac{1}{2}k_{1}l_{2}^{2}$$

(9) $$W_{2}{\left(l_{2}\right)}_{\lambda }=\frac{1}{2}k_{1}l_{1}^{2}+k_{1}l_{1}(l_{2}-l_{1})+\frac{1}{2}k_{2}{\left(l_{2}-l_{1}\right)}^{2}$$

It can be also seen from Figure 5 that *W*_2_(*l*_2_)_*λ*_, the out-of-displacement at *x* = *l*_2_, was larger than *W*_2_(*l*_2_)_*λ* = 1_ when *λ* > 1, otherwise it was smaller. Thus, in order to reduce the warpage of the beam, the *λ* < 1 had to be selected. That meant the beam with *k*_2_ < *k*_1_ was preferred. In addition, from Equation (9), the curvature *k*_1_ played a more important (or dominant) role than *k*_2_ in the value of *W*_2_(*l*_2_)_λ_ because it had an additional slope term *k*_1_*l*_1_(*l*_2_ − *l*_1_) affecting *W*_2_(*l*_2_)_*λ*_. For parametric demonstrations, the normalized *W*_2_(*l*_2_)_*λ*_ by the value of *W*_2_(*l*_2_)_*λ* = 1_ is shown as

(10) $$\frac{W_{2}{\left(l_{2}\right)}_{\lambda }}{W_{2}{\left(l_{2}\right)}_{\lambda =1}}={\left(\frac{l_{1}}{l_{2}}\right)}^{2}+\frac{2l_{1}(l_{2}-l_{1})}{l_{2}^{2}}+\frac{k_{2}}{k_{1}}\frac{{\left(l_{2}-l_{1}\right)}^{2}}{l_{2}^{2}}$$

The above equation could be further expressed in terms of *λ* and *β*, as described by

(11) $$\frac{W_{2}{\left(l_{2}\right)}_{\lambda }}{W_{2}{\left(l_{2}\right)}_{\lambda =1}}=\frac{2}{\beta }-\frac{1}{\beta^{2}}+\lambda {(1-\frac{1}{\beta })}^{2}$$

**Figure 6:**
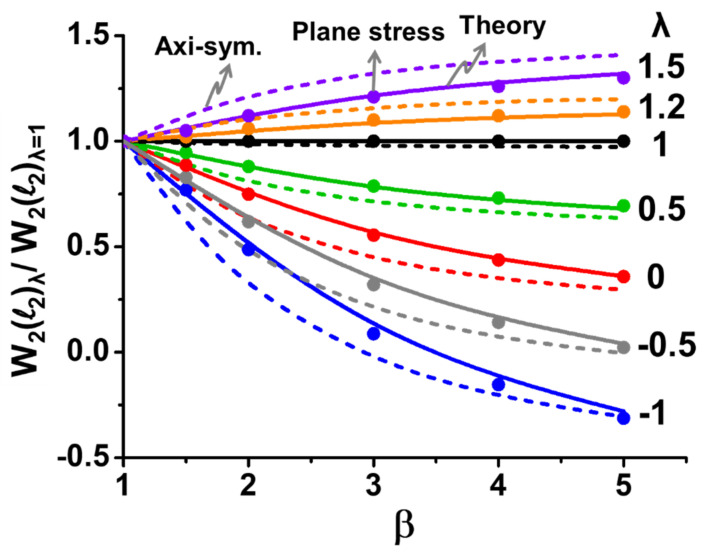
Normalized warpage (*W*_2_(*l*_2_)_*λ*_/*W*_2_(*l*_2_)_*λ*_ = _1_) vs. normalized beam length (*β* = *l*_2_/*l*_1_) for the beam theory (in solid lines) with various values of normalized curvature (*λ* = *k*_2_/*k*_1_) compared with the results from the plane stress (in dot points) and the axisymmetric models (in dash lines) of 2D FEM analyses.

**Figure 7:**
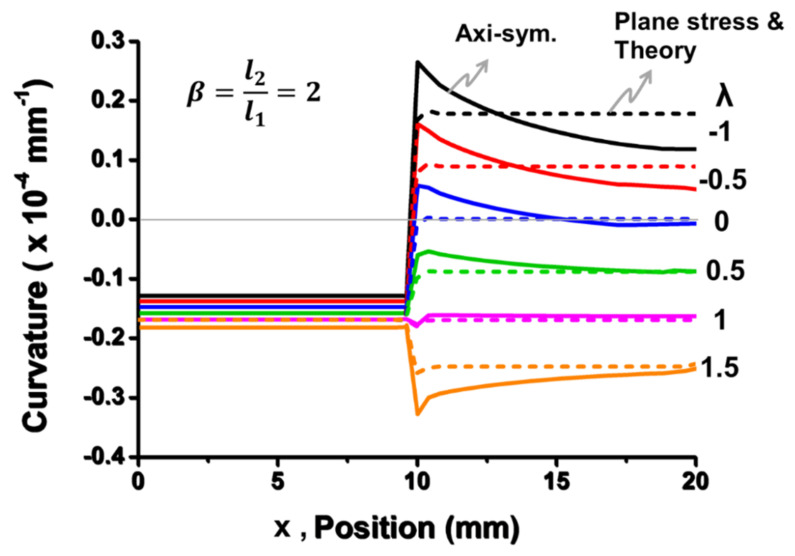
Curvature distributions along the length of the beam for the *β* = 2 case from the beam theory (in dash lines) with various values of normalized curvature (*λ*), compared with the results from plane stress (in dash lines) and axisymmetric models (in solid lines) of 2D FEM analyses.

**Figure 8:**
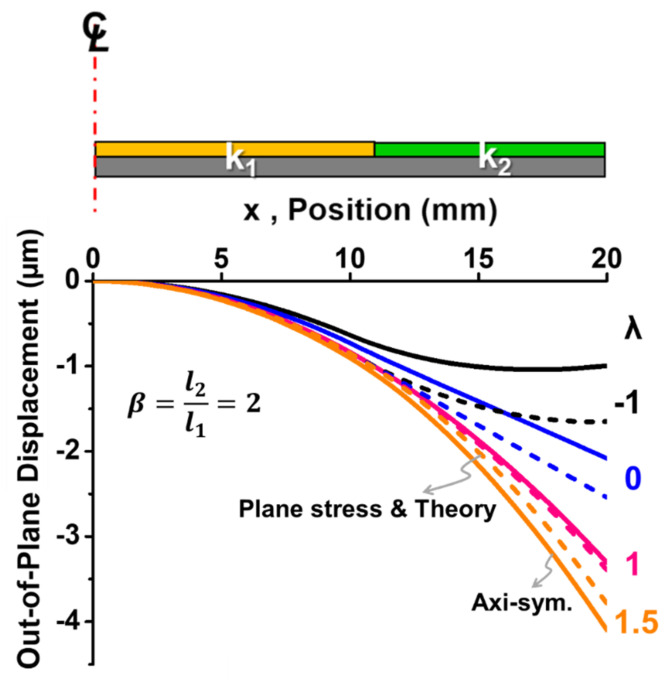
Out-of-plane displacement of the beam along the length of the beam for the *β* = 2 case from the beam theory (in dash lines) with various values of normalized curvature (*λ*) compared with the results from 2D plane stress (in dash lines) and axisymmetric (in solid lines) models of FEM.

For validation of this beam theory, Equation (11) is plotted against *β* and *λ* in Figure 6 and was compared with the results from the plane stress and the axisymmetric models of 2D FEM analyses. It is shown that the results from the beam theory and the plane stress model were very consistent, but they were slightly different from the axisymmetric model, which represented a plate (with a Poisson effect) rather than a beam (without a Poisson effect). Moreover, for *λ* = 1, the results from the beam theory and the plane stress model were close to those from the axisymmetric model; for *λ* < 1, they were overestimated, but for *λ* > 1, they were underestimated. In order to understand this mechanism, curvature distributions for the *β* = 2 case from the beam theory are plotted in Figure 7 along the entire length of the beam with various values of normalized curvature (*λ*) in comparison with those from the 2D plane stress and the axisymmetric models of the FEM analyses. It was found that, unlike consistent and constant curvatures (*k*_1_ and *k*_2_) over each segment between the beam theory and the plane stress model, the axisymmetric model (representing the plate case) gave smaller curvature *k*_1_ and non-uniform *k*_2_ values as *λ* < 1. In other words, the out-of-plane displacement (or warpage) caused by smaller curvature *k*_1_ and non-uniform *k*_2_ resulting from the Poisson effect in the axisymmetric model was lower than that in the beam theory and the plane stress model. As a result, both beam and plane stress models gave an overestimated value of the displacement or the warpage as *λ* < 1. However, for *λ* > 1, this was reversed. Furthermore, to examine the detailed warpages, the out-of-displacement of the beam along the entire length of the beam for the *β* = 2 case is plotted for those three models in Figure 8. It can be also seen that the out-of-plane displacement curves were very consistent for the three models for *λ* = 1, but the curves from both beam theory and plane stress model started deviating from those in the axisymmetric model with increasing values for *λ* < 1 and decreasing values for *λ* > 1. Based on the above observations, it was proven that the beam theory described in Equation (7) coincided with the 2D plane stress model but had some deviations from the 2D axisymmetric model due to the Poisson effect. The question is whether the beam theory can be used for calculating the out-of-displacement of the plate-like IC packages with the curvature data obtained from the strain gauge measurements. That is answered later in this paper.

(b) Validation of FEM simulation with shadow moiré

**Figure 9:**
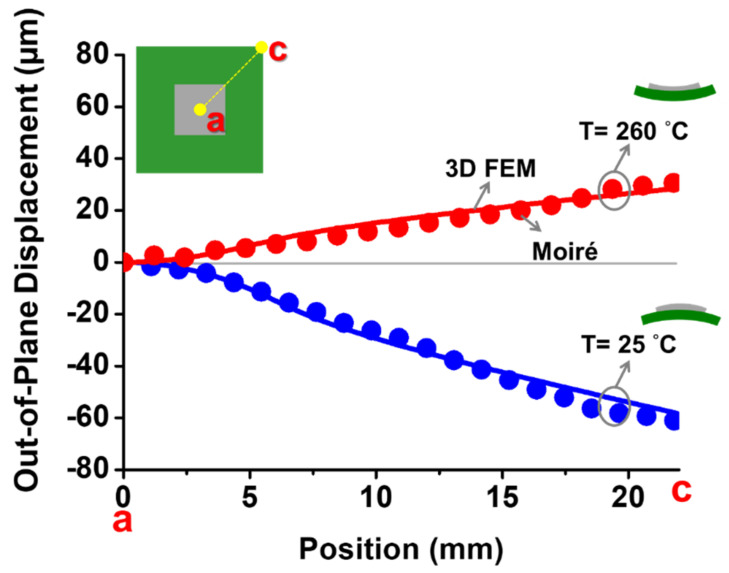
Out-of-plane displacement of the package along the diagonal line *ac* and with a full field at room temperature (T = 25 °C) and T = 260 °C from the 3D FEM analysis compared with moiré results.

To verify the result from the 3D FEM analysis, the out-of-plane displacements of the flip-chip package along the diagonal line *ac* at room temperature (T = 25 °C) and T = 260 °C are shown in Figure 9 from the 3D FEM analysis and the moiré measurement. It can be seen that the 3D FEM model effectively and precisely described the thermal deformation of the flip-chip package based on those consistent results. However, whether the 2D FEM (with axisymmetric model) or the strain gauge measurement associated with the beam model theory can do the same is discussed later.

(c) Implementation of gauge measurement

**Figure 10:**
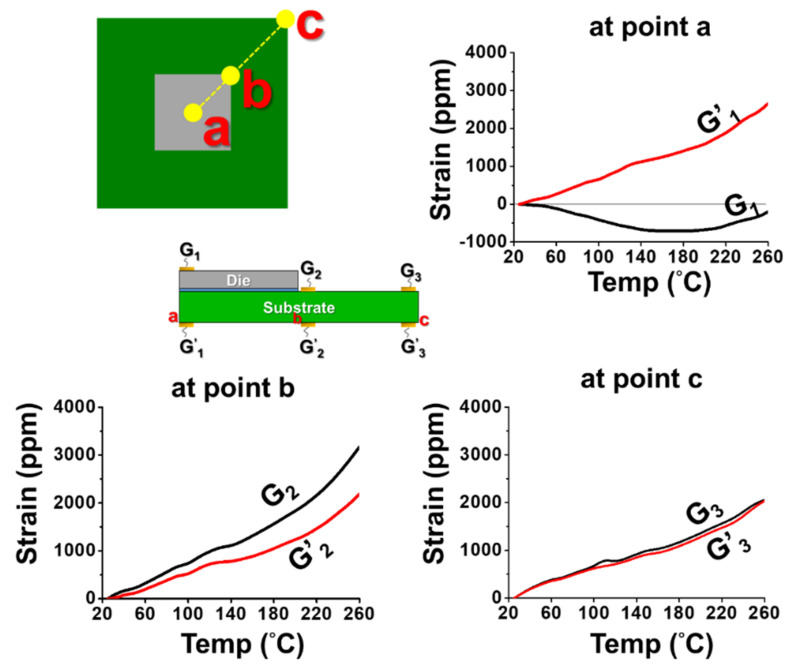
The strain data from the back-to-back gauges with various temperatures and with gauges G_1_/G_1′_, G_2_/G_2′,_ and G_3_/G_3′_ at the points *a*, *b*, and *c* (representing center point, near die corner, and substrate corner points), respectively, during the strain measurement under thermal loading from 25 °C to 260 °C in heating.

**Figure 11:**
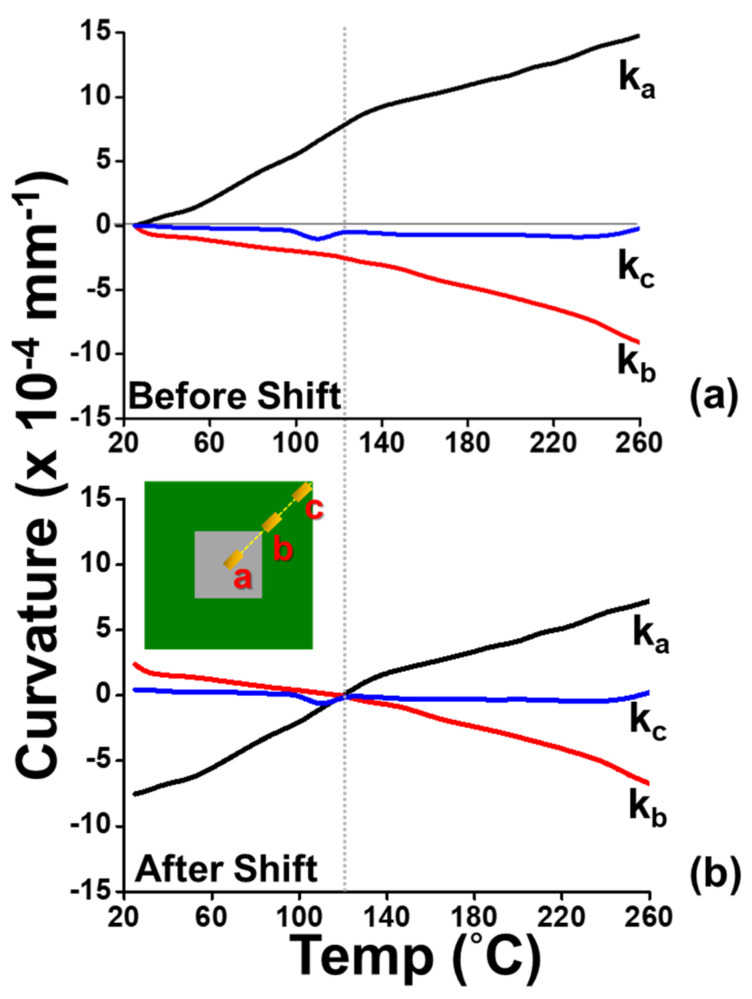
The curvature data at the points *a*, *b*, and *c* (representing center point, near die corner, and substrate corner points, respectively) with various temperatures from the strain gauge measurement (**a**) before shift and (**b**) after shift to 120 °C with the zero curvature.

**Figure 12:**
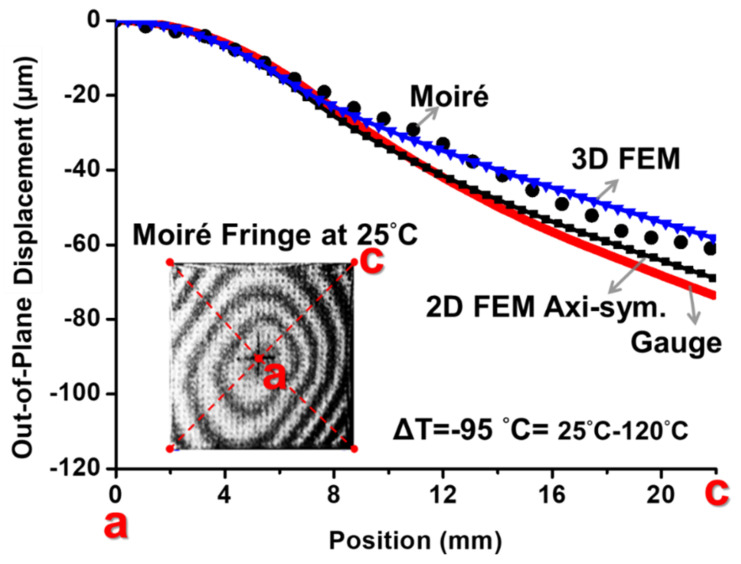
Out-of-plane displacement of the package at room temperature (T = 25 °C) along the diagonal line *ac* from the strain gauge measurement compared with the results from moiré data (with a sensitivity of 12.7 µm/fringe) and FEM results (with 2D axisymmetric and 3D models).

**Figure 13:**
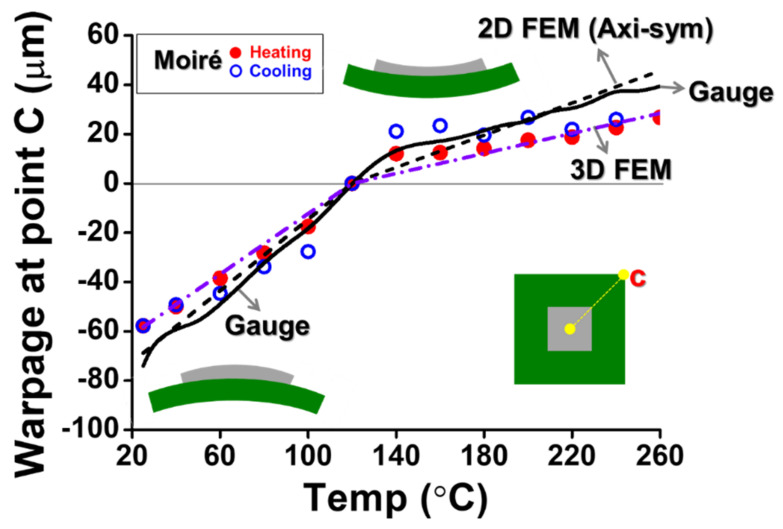
The thermally induced warpage of the package at the point c (the corner of the package) with various temperatures from the strain gauge measurement compared with the results from moiré data and FEM results (with 2D axisymmetric and 3D models).

The back-to-back gauges G_1_/G_1′_, G_2_/G_2′_, and G_3_/G_3′_ were attached on the top and the bottom surfaces of the flip-chip package at the points *a*, *b*, and *c* (representing center point, near die corner, and substrate corner points), respectively, for the strain measurement. The measured strain data with various temperatures are shown in Figure 10. The data indicate that the difference of the strain data between the bottom and the top gauges was large at point *a* (the center point), while it was small at point c (near substrate corner). That meant there existed the bending strains (*ε_b_*) with a large value at the center point, an intermediate value at the near die corner, and a small value near the substrate corner based on Equation (4). Furthermore, by Equation (5), those bending strain data could be converted to the curvature data, which are shown in Figure 11a, for various temperatures with curvatures *k_a_*, *k_b_*, and *k_c_* at the measured points *a*, *b*, and *c*, respectively. It was found that there existed a kinked point on the curve of *k_a_* but not on other curves near T = 120 °C, which was the glass transition temperature (T_g_) of the underfill material. This resulted from the elastic modulus (E) of the underfill material with about two orders of magnitude decrease at a temperature above its T_g_ [5]. Additionally, at this T_g_, the curvature on every point on the flip-chip package was almost zero based on the observation of flatness of the package in moiré experiments. Thus, all curvature curves could be further shifted to T = 120 °C with the predefined zero curvature (shown in Figure 11b). It was evident that there existed the apparent value of *k_b_* (near the corner of the chip) but with the close zero value of *k_c_* near the substrate corner. This was due to the Poisson effect, which was consistent with the case of λ = 0 with the axisymmetric model in Figure 7. Moreover, the curvature data from T = 120 °C cooling to 25 °C in Figure 11b were used to calculate the out-of-plane displacement of the package at room temperature (T = 25 °C) along the diagonal line *ac* using Equation (7), and this displacement result obtained from the strain gauge was compared with those from moiré and FEM (with 2D axisymmetric and 3D models), as shown in Figure 12. It was shown that, due to an inherent limitation of the beam theory (Equation (7)), the displacements obtained from strain gauge were closer to those of the 2D axisymmetric FEM model than those from moiré and 3D FEM models. Furthermore, the thermally induced warpages of the package at the point *c* (the corner of the package) from the strain gauge measurement are plotted in Figure 13 against temperature in comparison with those from moiré data and FEM results (with 2D axisymmetric and 3D models). Results also indicated that the gauge result was consistent with the 2D axisymmetric result but was slightly off from results of moiré and 3D FEM models. However, in the engineering applications, the strain gauge measurement associated with the beam model theory was accordingly proved to be feasible and good enough to determine the thermally induced out-of-plane deformations and warpage of the flip-chip packages.

## 4. The Case of 2.5D IC Package

(a) Validation of FEM simulation

**Figure 14:**
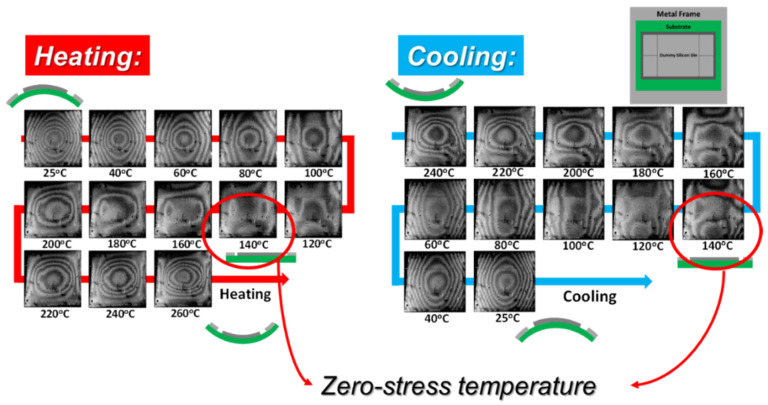
Moiré fringe patterns of the 2.5D IC package with a metal frame under thermal loading from 25 °C to 260 °C in heating and then from 260 °C to 25 °C in cooling (with a sensitivity of 25.4 µm/fringe) [16].

**Figure 15:**
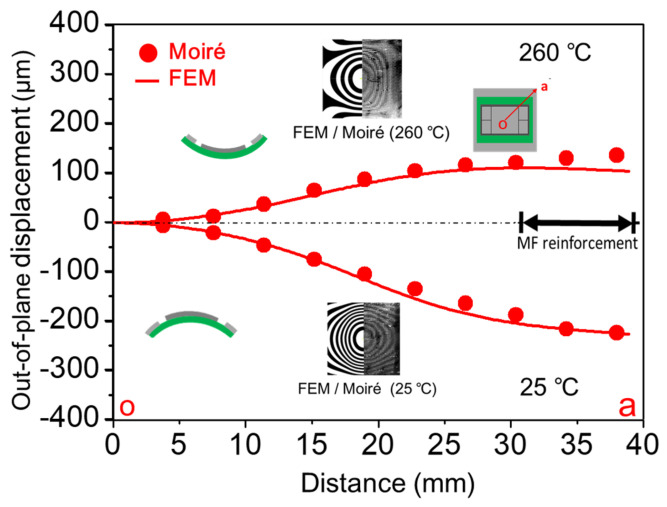
Out-of-plane deformation along the diagonal line *oa* for 2.5D IC package at the temperatures of 25 °C and 260 °C from moiré and FEM results.

Moiré measurement of the 2.5D IC package with a metal frame under thermal loading from 25 °C to 260 °C in heating and cooling was performed, and its moiré fringe patterns (out-of-plane displacement contours with a sensitivity of 25.4 µm/fringe) are shown in Figure 14 [16]. It was obvious that, during the heating process, the spherically convex shape of the specimen at 25 °C became less warped as the temperature increased and then turned out to be flat at 140 °C. Upon continuous heating, the specimen became concave above 140 °C and increasingly up to 260 °C. On the other hand, the specimen deformed in the reverse way during the cooling process and then came back to the same deformed shape at 25 °C. Such thermal elastic deformations of the package along the diagonal line *oa* at 25 °C and 260 °C are plotted and shown in Figure 15 in detail in comparison with those from the FEM analysis. The consistent results between moiré and FEM analysis indicated that the FEM model was valid and precise enough to describe the thermal deformations of the 2.5D IC package.

(b) Beam model verification

**Figure 16:**
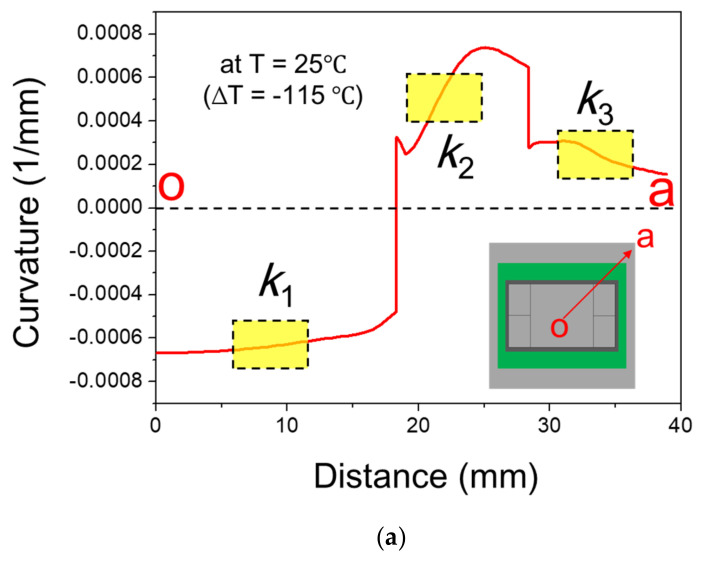

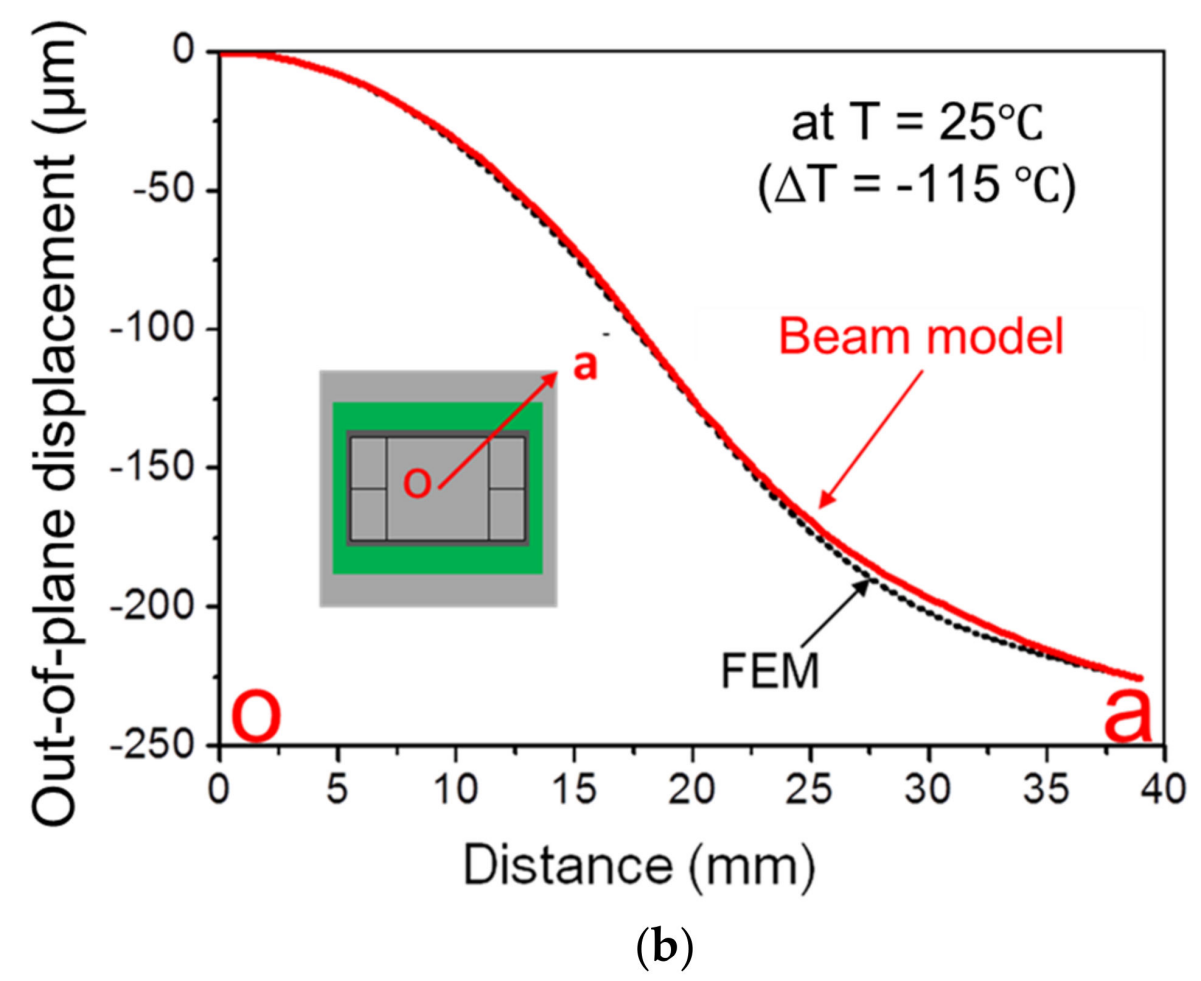
(**a**) Curvature distribution along the diagonal line *oa* of the 2.5D IC package and the average curvatures *k*_1_, *k*_2_, and *k*_3_ over the gauge length at each segment from FEM analysis, and (**b**) comparison of out-of-plane displacements of the 2.5D package from FEM and beam model associated with the average curvatures *k*_1_, *k*_2_, and *k*_3_.

After the FEM model was validated, this model was then employed for verifying the beam model theory, which was applied to calculate the thermal deformations of the 2.5D IC package with the strain gauge data later. Curvature distribution along the diagonal line *oa* of the package under thermal loading ΔT = −115 °C (from 140 °C cooling down to 25 °C) from the FEM analysis is shown in Figure 16a with the average curvatures of *k*_1_, *k*_2,_ and *k*_3_ over the gauge length at the corresponding segment. These gauge-assumed curvature data extracted from the FEM model were put into the beam model theory as described in Equation (7) to calculate the out-of-plane displacement. The thermally induced out-of-plane displacement of the 2.5D IC package obtained is shown in Figure 16b compared with that from the FEM analysis. The almost identical results of both methods revealed that the method of using the point-wise data of strain gauge associated with the beam model theory was feasible for measuring the thermal deformation of the 2.5D IC package.

(c) Implementation of strain gauge measurement

**Figure 17:**
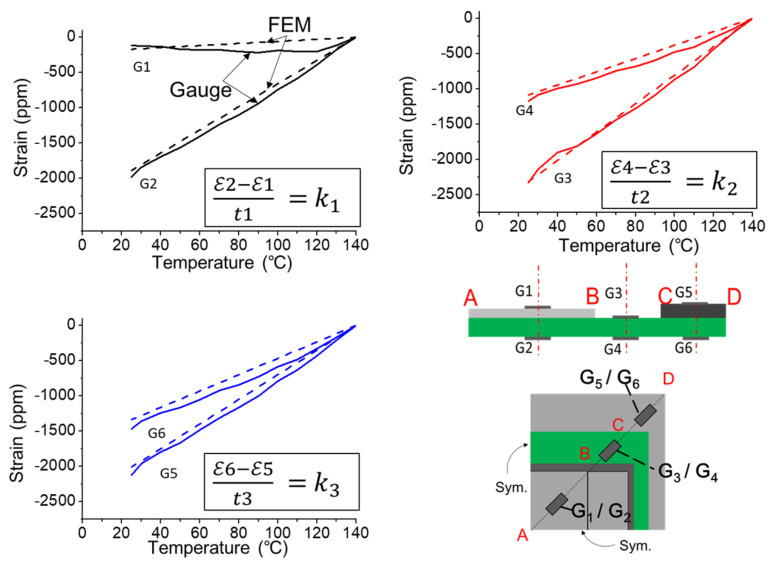
Axial strains from gauge measurements and FEM simulation along the diagonal line of the 2.5D IC package under thermal loading from 140 °C to 25 °C in cooling.

**Figure 18:**
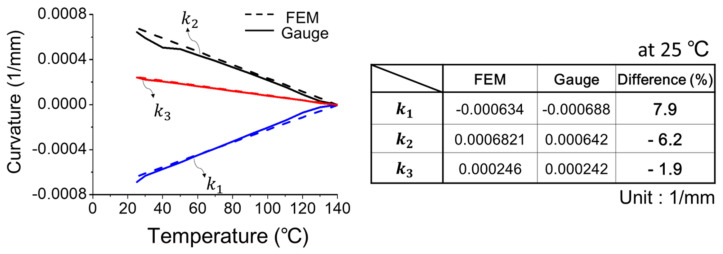
Comparison of the curvature data (*k*_1_, *k*_2_, and *k*_3_) extracted from strain gauge measurement and FEM simulation for the 2.5D IC package at various temperatures.

**Figure 19:**
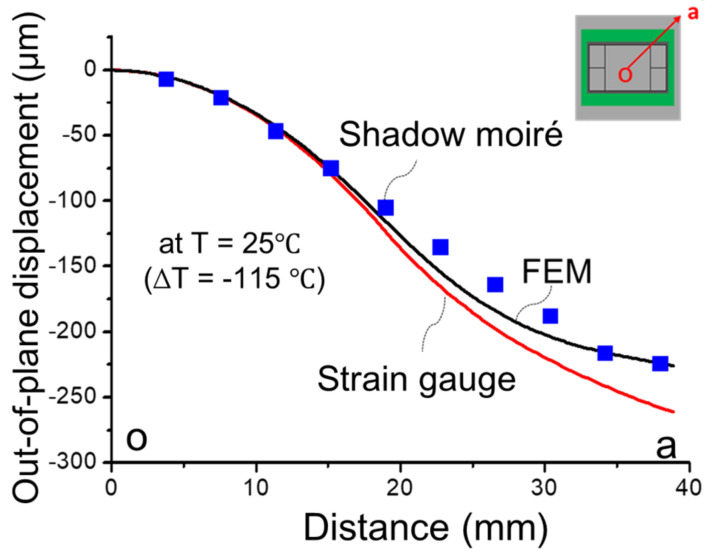
Comparison of the out-of-plane displacement of the 2.5D IC package at temperature of 25 °C (∆T = −115 °C) from strain gauge measurement, shadow moiré, and FEM simulation.

**Figure 20:**
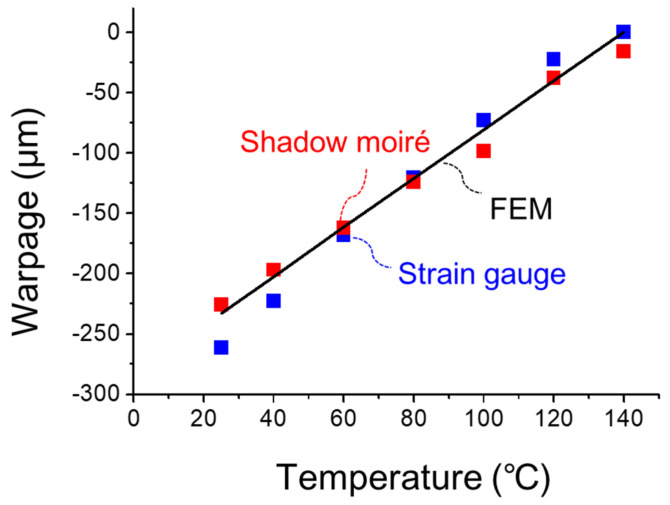
Comparison of warpages of the 2.5D IC package at various temperatures from strain gauge measurement, shadow moiré, and FEM simulation.

The strain gauge measurement was further carried out for determining the bending curvatures and deformations of the 2.5D IC package under thermal loading from 140 °C to 25 °C in the cooling process. Note that the strain gauge readings were reset at zero at 140 °C. In Figure 17, the back-to-back strain gauges were attached at the centers of lines *AB*, *BC*, and *CD* along half of a diagonal line with the gauge pairs of G1/G2, G3/G4, and G5/G6, respectively. The axial thermal strains of each reading from those six gauges are also shown in Figure 17 in comparison with those from the FEM simulation. The consistency between both data indicated that the strain gauges could measure the thermal strains of the package. The bending curvature data under this thermal loading were further extracted using Equations (4) and (5) and are shown in Figure 18 in a detailed comparison with the FEM results. Almost identical results revealed that the curvature data for *k*_1_, *k*_2_, and *k*_3_, corresponding to those on lines *AB*, *BC*, and *CD*, increased positively for *k*_2_ and *k*_3_ but increased negatively for *k*_1_ in the cooling process. Their maximum curvatures occurred at 25 °C. The differences of the maximum curvatures between strain gauge measurement and FEM simulation were within 8% (listed in the left table). Moreover, the out-of-plane displacement at 25 °C was plotted using Equation (7) of the beam model theory associated with the curvature data (*k*_1_, *k*_2_, and *k*_3_) from the gauge measurement and is shown in Figure 19 in comparison with those from shadow moiré measurement and FEM simulation. The results indicated that the out-of-plane displacement of the 2.5D IC package from gauge measurement reasonably agreed with the other two with a minor difference. The warpages of the 2.5D IC package at various temperatures are further plotted in Figure 20 from the strain gauge measurement, the shadow moiré measurement, and the FEM simulation. Those results also showed a reasonably good agreement between each other. Overall, this study demonstrated that the strain gauge measurement associated with the beam model theory can be used for characterizing the out-of-plane thermal deformation and warpage of the 2.5D IC package.

(d) Effect of segment curvature variation on warpage

It is interesting to know how the curvature data *k*_1_, *k*_2_, and *k*_3_ at each segment affected the warpage of the 2.5D IC package. The plus and the minus 10% variations of each curvature data in Figure 16a used in plotting displacement using the beam model were analyzed, and the obtained warpage results and their difference are listed in Table 2. Note that *k*_1_, *k*_2_, and *k*_3_ were normalized values and defined as unity for reference. It could be seen that the warpage was the most sensitive to *k*_1_, rather than *k*_2_ and *k*_3_, with increases or decreases of ~15% of warpage value by changing ±10% of *k*_1_ values, respectively. This was consistent with the above-mentioned finding that *k*_1_, the curvature of the center segment, was a dominant value in the warpage of the packages. Therefore, the precisely defined or measured *k*_1_ was more important than the other two (*k*_2_ and *k*_3_) for the warpage calculation or the measurement of the 2.5D IC package.

(e) Effect of gauge misalignment on warpage

Since the precise measurement of *k*_1_ was critical for determining the warpage of the 2.5D IC package, it was desired to understand how the misalignment of *k*_1_ gauges affected the warpage. Various angle misalignments (with ±5° and ±10°) of top and bottom strain gauges in the 45° direction in segment *AB* (the center segment) were analyzed using a beam model for calculating the package warpage through the measurement of *k*_1_ curvatures. The results are shown in Table 3, in which the warpage determined from the top and the bottom gauges in the angle of 45° was used as a based (or reference) value. It could be seen that, for ±5° misalignments, the obvious warpage differences occurring at the angle pairs of 50°/50° and 40°/40° were 3.57% and −3.76%, while the other pairs were below 3%. However, for ±10° misalignments, the warpage difference increased by more than double. As a result, as long as the gauge alignment on the top and the bottom surfaces was under control with less than ±5° misalignments, the results of the warpage measurement were acceptable with less than 5% error.

## 5. Conclusions

This study proposed the strain gauge method associated with a beam model theory for determining the thermally induced deformations and warpages of the flip-chip and the 2.5D IC packages. The beam model theory was thoroughly evaluated in this study, and it was found that the theory coincided with the 2D plane stress model but had some deviations from the 2D axisymmetric model due to the Poisson effect. The finite element method (FEM) resulted with good consistency with the full-field shadow moiré experiment, showing good prediction of thermally induced out-of-plane deformations for both packages. Furthermore, the strain gauge measurement with the beam model theory was actually implemented for both packages. It was found that the obtained thermal strain data were in good agreement with the FEM data. The gauge-determined thermal deformations and warpages of both packages also showed reasonably good agreement with those from the FEM analyses and the shadow moiré measurements. Moreover, the curvature in the center segment of the 2.5D IC package was found to be a dominant value in control of thermal warpages of the package. The gauge misalignment effect was also evaluated and discussed in detail. Overall, it was proven that the strain gauge method of featuring point strain measurement associated with the beam model theory can be feasible for measuring the thermal deformations and warpages of both packages.

## Figures and Tables

**Table 1:** Material mechanical properties for flip-chip and 2.5D IC packages used in the finite element analysis and provided by material vendors (* note that the effective composite material properties were used based on the rule of mixture).

Package Type	Material	E (MPa)	*ν*	α (ppm/°C)	Tg (°C)
Flip-chip Package	Si	131,000	0.28	2.8	-
	Bump/UF *	7600/100	0.32	29/98	120
	Substrate	26,000	0.39	14.4	-
2.5D IC Package	Si	169,000	0.28	2.3	-
	EMC	23,000/2300	0.69	10.8/24	140
	μBump/UF1 *	7200/72	0.28	28/97	140
	C4 Bump/UF2 *	7600/76	0.28	29/98	140
	Substrate	20,000	0.42	13.2	
	Metal Frame	117,000	0.3	16.9	
	Adhesive	100/20	0.3	125/170	50

**Table 2:** The warpage difference of the 2.5D IC package with a variation of segment curvatures at each segment.

**k_1_**	1	1.1	1	1	0.9	1	1
**k_2_**	1	1	1.1	1	1	0.9	1
**k_3_**	1	1	1	1.1	1	1	0.9
**Warpage (μm)**	−226	−260	−215	−224	−191	−236	−227
**Difference (%)**	0	15.34	−4.75	−0.60	−15.35	4.75	0.60

**Table 3:** The warpage (with a unit of µm) and the warpage difference of the 2.5D IC package with various angle misalignments (±5° and ±10°) of top and bottom strain gauges for measuring *k*_1_ curvatures in segment *AB* (the center segment).

**±5°**	**Top Strain Gauge**			
Bottom strain gauge	Degree	50°	45°	40°
	50°	−234 (3.57%)	−231 (2.38%)	−229 (1.3%)
	45°	−228 (0.96%)	−226 (Reference)	−223 (−1.31%)
	40°	−222 (1.49%)	−220 (−2.68%)	−217 (−3.76%)
**±10°**	**Top Strain Gauge**			
Bottom strain gauge	Degree	55°	45°	35°
	55°	−243 (7.49%)	−237 (5.06%)	−233 (3.02%)
	45°	−231 (2.21%)	−226 (Reference)	−221 (−2.26%)
	35°	−220 (−2.48%)	−215 (−4.91%)	−210 (−6.95%)

## Data Availability

On inquiry, the data presented in this study is available from the authors.
